# Real-World Evaluation of a Nationally Mandated Risk Stratification Tool for Destination Therapy LVAD in Japan

**DOI:** 10.1016/j.jacasi.2026.06.017

**Published:** 2026-07-24

**Authors:** Teruhiko Imamura, Koichiro Kinugawa, Takashi Nishimura, Koichi Toda, Yoshikatsu Saiki, Hiroshi Niinami, Shinichi Nunoda, Goro Matsumiya, Hirokuni Arai, Masanobu Yanase, Norihide Fukushima, Takeshi Nakatani, Akira Shiose, Ikuko Shibasaki, Yasushi Sakata, Masaru Hatano, Minoru Ono, J-MACS Investigators

**Affiliations:** a Second Department of Internal Medicine, University of Toyama, Toyama, Japan; b Department of Cardiovascular and Thoracic Surgery, Ehime University, Matsuyama, Japan; c Department of Cardiovascular Surgery, Dokkyo Medical University Saitama Medical Center, Saitama, Japan; d Division of Cardiovascular Surgery, Tohoku University, Sendai, Japan; e Department of Cardiovascular Surgery, Tokyo Women's Medical University, Tokyo, Japan; f Department of Therapeutic Strategy for Severe Heart Failure, Tokyo Women's Medical University, Tokyo, Japan; g Department of Cardiovascular Surgery, Chiba University Graduate School of Medicine, Chiba, Japan; h Department of Cardiovascular Surgery, Tokyo Medical and Dental University, Tokyo, Japan; i Department of Cardiology, Fujita Health University Bantane Hospital, Nagoya, Japan; j Department of Transplantation, National Cerebral and Cardiovascular Medicine, Suita, Japan; k Senri Kinran University, Suita, Japan; l Maki Hospital, Osaka, Japan; m Department of Cardiovascular Surgery, Kyushu University, Fukuoka, Japan; n Department of Cardiovascular Surgery, Iwaki City Medical Center, Fukushima, Japan; o Department of Cardiovascular Medicine, Osaka University, Suita, Japan; p Department of Cardiology, University of Tokyo, Tokyo, Japan; q Department of Cardiac Surgery, University of Tokyo, Tokyo, Japan

**Keywords:** heart failure, hemodynamics, mechanical circulatory support, mortality

## Abstract

**Background:**

Patient selection for destination therapy (DT) with durable left ventricular assist devices (LVADs) remains a major challenge in Asian advanced heart failure practice. In Japan, the J-MACS score is mandatorily calculated before DT implantation and directly influences eligibility decisions; however, its real-world prognostic performance in DT recipients remains unclear.

**Objectives:**

The authors aimed to externally validate the predictability of the J-MACS score on mortality in a nationwide Japanese DT cohort.

**Methods:**

We conducted a nationwide registry-based analysis of patients undergoing HeartMate 3 implantation as DT between May 2021 and October 2025. The primary endpoint was 2-year all-cause mortality. Prognostic performance of the J-MACS score—based on age, prior cardiac surgery, serum creatinine, and central venous pressure–to–pulmonary artery wedge pressure ratio—was evaluated using multivariable Cox models and restricted mean survival time.

**Results:**

In total, 210 patients (median age, 60 years; 159 [76%] male) were followed for a median of 514 days (IQR: 257-730 days) after LVAD implantation. The J-MACS score independently predicted mortality (adjusted hazard ratio: 1.17, 95% CI: 1.02-1.36, *P* = 0.037). The 2-year cumulative mortality rates were 5.8% (95% CI: 2.0%-16.1%) in the low-risk group, 12.7% (95% CI 6.3%-24.6%) in the intermediate-risk group, and 28.6% (95% CI 14.6%-51.2%) in the high-risk group (*P* = 0.003 for comparison). The high-risk group had approximately 4 months shorter survival.

**Conclusions:**

This nationwide real-world study provides the first validation of a nationally mandated DT eligibility algorithm. The J-MACS score may support risk-based clinical decision-making in Japanese DT practice.

Heart failure remains a major global health challenge, with rising prevalence, repeated hospitalizations, and persistently poor long-term outcomes despite substantial advances in pharmacological and device-based therapies.^1^^,^^2^ For patients with end-stage heart failure refractory to optimal medical therapy, durable left ventricular assist device (LVAD) implantation has become an established life-prolonging treatment, if applicable.^3^^,^^4^

LVAD therapy has primarily been used as a bridge to heart transplantation. However, with improvements in device design, durability, and management strategies, destination therapy—defined as permanent mechanical circulatory support for patients ineligible for transplantation—has gained wide acceptance in North America and Europe.^5^

In Asian countries, including Japan, limited donor availability and distinct health care system structures have shaped unique patient selection strategies for advanced therapies. In Japan, destination therapy with the HeartMate 3 LVAD was officially approved in May 2021, representing a major advance in the management of advanced heart failure patients who are not transplant candidates.^6^ While this expansion of therapy offers new hope, achieving acceptable outcomes requires careful patient selection, given the high-risk nature and cost of long-term LVAD support. Accordingly, accurate preoperative risk stratification tools are essential for improving clinical outcomes.^7^

Several risk-prediction models, including the HeartMate II Risk Score (HMII-RS) and HeartMate 3 Risk Score (HM3-RS), have been proposed and validated in Western populations.^8^, ^9^, ^10^ However, these models were derived predominantly from non-Asian cohorts and may not be directly applicable to Japanese patients, whose demographic and clinical profiles differ considerably.^6^ To address this limitation, the J-MACS (Japanese registry for Mechanically Assisted Circulatory Support) score was created using nationwide J-MACS registry data^11^ on patients who received durable LVAD implantation as a bridge to transplantation, aiming to provide a simple and objective preoperative risk assessment tool.^12^ In this study, the predictability of 3-year mortality was found to be superior with the J-MACS score compared to other conventional risk scores.^12^ After the development/publication of this score, it has become mandatory to calculate the J-MACS score before LVAD implantation as destination therapy in Japan. In principle, patients with low- or intermediate-risk are considered eligible for destination therapy. The applicability of the J-MACS score has been validated in various other clinical scenarios.^13^^,^^14^

However, despite its mandatory incorporation into clinical practice, the prognostic performance of the J-MACS score has not been validated in real-world destination therapy recipients since the approval of the HeartMate 3. Therefore, this study aimed to evaluate for the first time the real-world clinical validity of this nationally implemented risk-based therapeutic framework, without any modification, using data on the destination therapy cohort in the J-MACS registry and comparing the predictability with other conventional scores.

## Methods

### Patient selection

Patients who underwent implantation of a standard durable LVAD at participating Japanese institutions were enrolled in the prospectively maintained J-MACS registry database.^11^ Among them, those who received durable LVAD implantation as destination therapy were included in this retrospective analysis.^6^

In Japan, the HeartMate 3 was approved for destination therapy in May 2021. Therefore, all patients in the present study underwent HeartMate 3 implantation between May 2021 and October 2025. Patients were followed for 2 years after implantation or until October 2025, whichever came first. Individuals who received durable LVADs for other indications, including bridge to transplantation, were excluded, as were those with critical missing data.

All participants provided written informed consent before surgery at each participating center. The use of the J-MACS registry for this analysis and the study protocol were approved by the J-MACS Committee on October 21, 2025. All procedures were conducted in accordance with the principles outlined in the Declaration of Helsinki.

### Study design

This retrospective external validation study included patients who received HeartMate 3 LVAD as destination therapy between May 2021 and October 2025 at all certified institutions in Japan. Patients were followed for 2 years after implantation or until the end of the study period (October 2025). Day 0 was defined as the date of LVAD implantation.

The primary outcome was all-cause mortality within 2 years. The J-MACS score, which consists of 4 baseline variables—age, history of cardiac surgery, serum creatinine level, and a central venous pressure–to–pulmonary artery wedge pressure (CVP/PAWP) ratio >0.71—was used without modification and calculated for all participants.^12^ Based on this score, patients were categorized into low-, intermediate-, and high-risk groups using predefined cutoff values of 10.2 and 13.7, respectively, which were reported in the original derivation study. The prognostic performance of the J-MACS score for predicting the primary outcome was evaluated.

### Preoperative baseline data

Standard preoperative clinical data were collected to evaluate patients' baseline characteristics, including demographic information, comorbidities, laboratory findings, echocardiographic parameters, hemodynamic measurements, and medication profiles. A history of cardiac surgery was defined as any prior coronary artery bypass grafting or valvular surgery.

### Calculation of risk scores

The J-MACS score was calculated using baseline data obtained before LVAD implantation according to the following formula: 0.105 × (age in years) + 2.06 × (history of cardiac surgery) + 3.56 × (serum creatinine [in mg/dL]) + 2.61 (if CVP/PAWP ratio >0.71).^12^ Other previously proposed scores were also calculated as follows.

(1) HMII-RS was calculated using the following formula, which was developed by modifying the original formula^8^ to be more suitable for the Japanese cohort: 0.0274 × (age in years) – 0.723 × (serum albumin [in g/dL]) + 0.74 × (serum creatinine [in mg/dL]) + 1.136 × (international normalized ratio of prothrombin time) + 0.807 × (1 for centers with <3 implants per 2 years and 0 for centers with ≥3 implants per 2 years).^6^
(2) HM3-RS was calculated using the following formula: 0.03496 × (age in years) + 0.53029 (if previous coronary artery bypass grafting or valve surgery) – 0.04112 × (serum sodium [in mEq/L] – 136) + 0.01093 × (blood urine nitrogen [in mg/dL]) + 0.62149 (if left ventricular end-diastolic diameter <5.5 cm) + 0.44785 (if CVP/PAWP ratio >0.6).^9^

### Follow-up protocol

All patients received guideline-directed medical therapy, including aspirin and warfarin, with a target international normalized ratio of 2.0 to 2.5 after LVAD implantation.^3^ Medication doses were adjusted according to the occurrence of bleeding or thromboembolic events. Detailed management protocols were determined at the discretion of each participating center. Device speeds were appropriately adjusted based on each patient's hemodynamic and echocardiographic findings.

### Statistical analyses

All statistical analyses were performed using SPSS Statistics version 23 (SPSS Inc). Two-sided *P*-values <0.05 were considered statistically significant. Continuous variables are presented as medians with interquartile ranges and were compared using the Mann-Whitney *U* test, whereas categorical variables are expressed as counts and percentages and were compared using the Fisher's exact test. Standardized mean differences were calculated to assess the balance of baseline characteristics between included and excluded patients, with a value <0.10 considered indicative of negligible imbalance.

The prognostic impact of the J-MACS score on 2-year mortality was evaluated using Cox proportional hazards regression, treating the score as a continuous variable. Prespecified potential confounders were assessed in univariable analyses, and variables with *P* < 0.10 were entered into multivariable models using forced entry. The proportional hazards assumption was assessed using Schoenfeld residuals. Model calibration was assessed by comparing predicted versus observed mortality using the observed-to-expected ratio for weak, the calibration slope for moderate, and the calibration plot for strong calibration, as well as the Hosmer-Lemeshow test. Fine-Gray subdistribution hazard analysis was additionally performed by assuming device explantation as a competing event. Missing values were handled using multiple imputation by chained equations (5 datasets). The imputation model included all baseline variables and the outcome variable. Continuous and binary variables were imputed using linear and logistic regression models, respectively. Analyses were performed separately within each dataset and combined using Rubin's rules. The adequacy of the imputation was assessed by comparing distributions of observed and imputed values and by confirming consistency of estimates across datasets. Multiplicative interaction analyses were performed by including an interaction term in the Cox proportional hazards model to evaluate whether the prognostic impact of the J-MACS score differed according to age category (>65 vs ≤65 years).

Risk group comparisons were performed using predefined J-MACS cutoffs, with survival differences assessed by Kaplan-Meier analysis and the log-rank test. Time-dependent receiver operating characteristic analysis was used to determine discrimination and optimal cutoff performance. Internal validation was performed using bootstrap resampling (1,000 iterations). The optimism-corrected C-statistic was calculated by subtracting the estimated optimism from the apparent C-statistic. Restricted mean survival time (RMST) over 2 years (730 days) was estimated using Kaplan-Meier methods, with differences evaluated by nonparametric bootstrap resampling (2,000 iterations).

Comparative analyses with HMII-RS and HM3-RS included Cox regression, log-rank testing, and comparison of Harrell's C-statistics using bootstrap-based methods. The clinical utility of the model was assessed by the decision curve analysis. Incremental prognostic utility was assessed using continuous net reclassification improvement (NRI) and integrated discrimination improvement (IDI).

## Results

### Baseline characteristics

A total of 264 patients underwent HeartMate 3 LVAD implantation as destination therapy between May 2021 and October 2025 and were registered in the J-MACS database.^11^ After excluding 54 patients with critical missing data (eg, CVP/PAWP ratio), 210 patients were included. All baseline characteristics were well balanced between included and excluded patients, with standardized mean differences <0.10 for all variables.

Baseline characteristics before LVAD implantation are summarized in Table 1. The median age was 60 years (IQR: 50-65 years), and 57 of the 210 patients (27%) were aged 65 years or older. Most patients were male (159/210; 76%). There were 64 patients (31%) with a history of cardiac surgery, and only 9 patients (4%) with a history of malignancy. The median serum creatinine level was 1.05 mg/dL (IQR: 0.76-1.37 mg/dL), and the common logarithm of plasma B-type natriuretic peptide concentration was 2.72 pg/mL (IQR: 2.48-2.86 pg/mL). The median CVP was 7 mm Hg (IQR: 5-11 mm Hg), and the PAWP was 19 mm Hg (IQR: 12-26 mm Hg). Only 15% of patients had a CVP/PAWP ratio >0.71. All patients received guideline-directed medical therapy before implantation.

**Table 1:** Baseline Characteristics of the Study Patients (N = 210)

Demographics	
Age, y	60 (50-65)
Age ≥65 y	57 (27%)
Male	159 (76%)
Body height, cm	168 (160-172)
Body weight, kg	58.9 (50.3-66.7)
Body surface area, m^2^	1.65 (1.53-1.77)
Systolic blood pressure, mm Hg	84 (77-94)
Diastolic blood pressure, mm Hg	57 (51-65)
Pulse rate, beats/min	80 (70-89)
Comorbidity	
History of cardiac surgery	64 (31%)
Diabetes mellitus	72 (34%)
Ischemic etiology	61 (29%)
Chronic obstructive pulmonary disease	3 (1%)
Peripheral artery disease	3 (1%)
History of stroke	24 (11%)
History of malignancy	9 (4%)
Cardiac resynchronization therapy	75 (36%)
Laboratory data	
Hemoglobin, g/dL	11.2 (9.8-12.5)
Serum albumin, g/dL	3.5 (3.1-3.9)
Serum total bilirubin, mg/dL	0.7 (0.5-1.1)
Serum sodium, mEq/L	137 (135-139)
Serum potassium, mEq/L	4.3 (4.0-4.5)
Serum blood urea nitrogen, mg/dL	19 (13-26)
Serum creatinine, mg/dL	1.05 (0.76-1.37)
C-reactive protein, mg/dL	0.34 (0.10-1.28)
Plasma B-type natriuretic peptide, common logarithm, pg/mL	2.72 (2.48-2.86)
Prothrombin time-international normalized ratio	1.2 (1.1-1.3)
Serum C-reactive protein, mg/dL	0.39 (0.12-1.76)
Echocardiography data	
Left ventricular end-diastolic diameter, mm	65 (57-73)
Left ventricular ejection fraction <40%	198 (94%)
Left atrial diameter, mm	45 (41-52)
Moderate or greater mitral regurgitation	115 (55%)
Moderate or greater tricuspid regurgitation	68 (32%)
Moderate or greater aortic regurgitation	30 (14%)
Hemodynamics	
Central venous pressure, mm Hg	7 (5-11)
Systolic pulmonary artery pressure, mm Hg	37 (27-49)
Diastolic pulmonary artery pressure, mm Hg	19 (13-25)
Mean pulmonary artery pressure, mm Hg	26 (18-34)
Pulmonary artery wedge pressure, mm Hg	19 (12-26)
Central venous pressure/pulmonary artery wedge pressure ratio	0.42 (0.27-0.59)
Cardiac output, L/min	3.55 (2.90-4.40)
Pulmonary artery pulsatility index	2.5 (1.6-4.0)
Pulmonary vascular resistance, Wood units	1.57 (0.81-2.66)
J-MACS score	10.2 (8.8-11.9)
Low/Intermediate/High score, n	106/77/27
Medications	
Intravenous inotropes use	165 (79%)
Beta-blocker use	182 (87%)
Renin-angiotensin system inhibitor use	136 (65%)
Mineralocorticoid receptor antagonist use	179 (86%)
Loop diuretics use	159 (76%)

Clinical data obtained before LVAD implantation are listed. Continuous variables are presented as median (IQR), and categorical variables are presented as n (%) unless otherwise indicated.

### Calculation of J-MACS score

The J-MACS score was calculated for all patients using 4 baseline clinical variables. The distribution of J-MACS scores varied widely, with a median value of 10.2 (IQR: 8.8-11.9) (Figure 1). Patients with scores <10.2 were assigned to the low-risk group (n = 106), those with scores between 10.2 and 13.7 to the intermediate-risk group (n = 77), and those with scores >13.7 to the high-risk group (n = 27).

**Figure 1:**
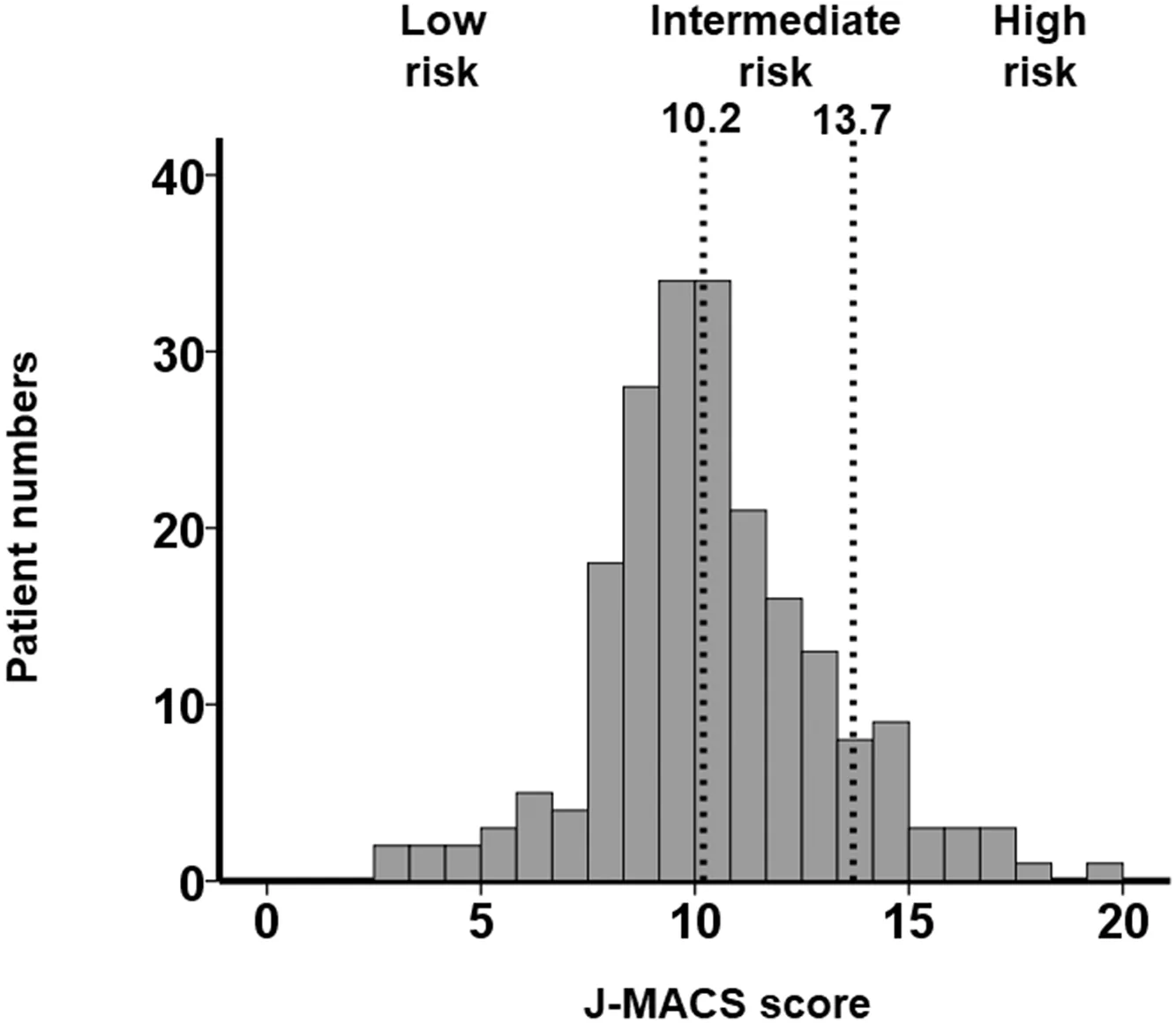
Distribution of the J-MACS Score The J-MACS score was calculated in all patients using 4 simple baseline characteristic data obtained before HeartMate 3 implantation. Patients were stratified into low-, intermediate-, and high-risk groups using the predefined cutoffs of 10.2 and 13.7.

The average contribution of each score component (age, history of cardiac surgery, serum creatinine, and CVP/PAWP ratio >0.71) across the 3 risk groups is shown in Figure 2. The average scores for age, history of cardiac surgery, and serum creatinine increased significantly with higher risk categories (all *P* < 0.001). In contrast, the average score for the CVP/PAWP ratio decreased significantly across the incremental risk groups (*P* = 0.002).

**Figure 2:**
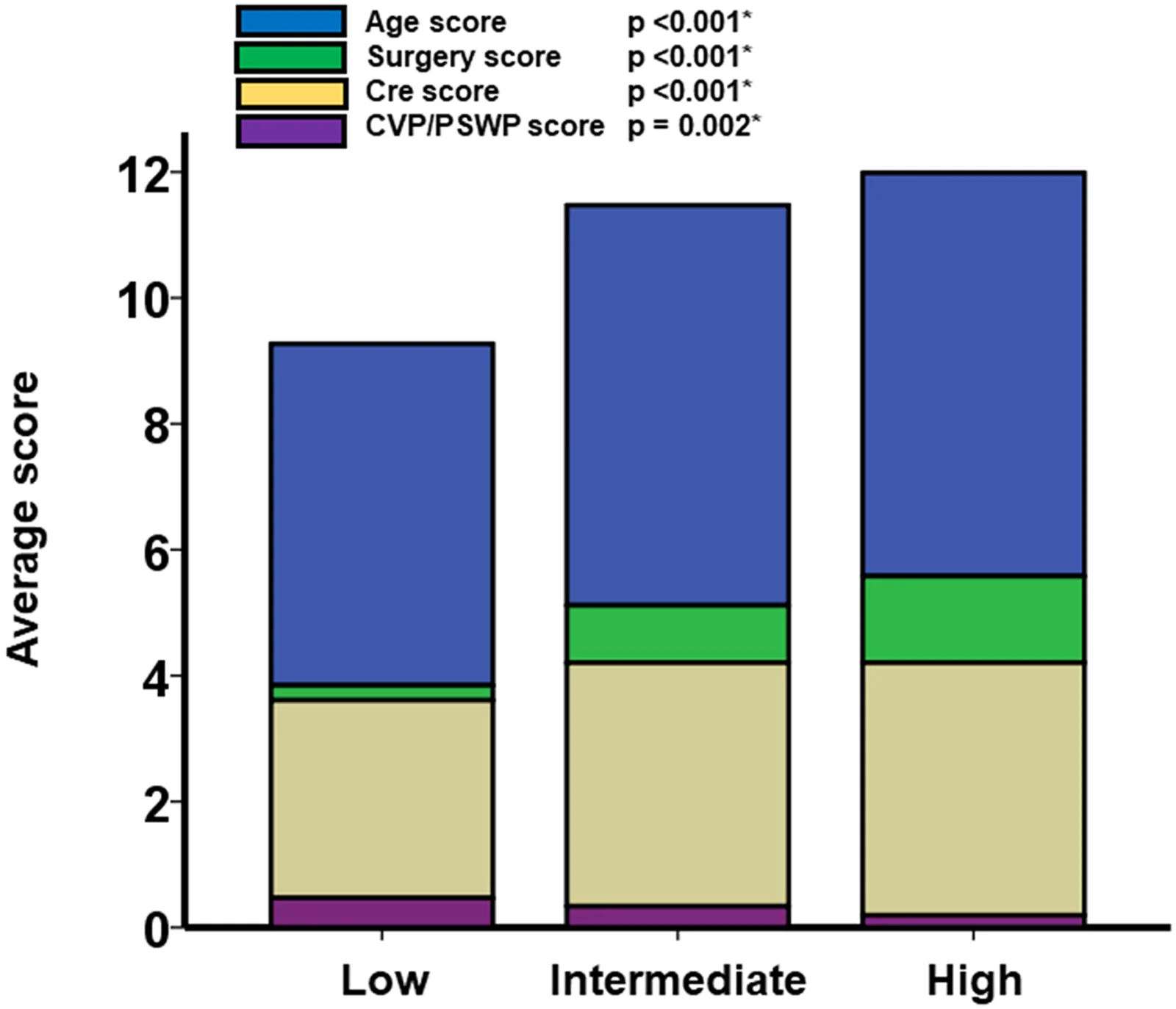
Comparison of the 4 Component Variables of the J-MACS Score Average scores for age, prior cardiac surgery, and serum creatinine increased progressively from low to high risk, whereas the CVP/PAWP ratio score was lower in higher-risk categories (∗*P* < 0.05 by Wilcoxon signed-rank test). Cre = creatinine; CVP = central venous pressure; PAWP = pulmonary artery wedge pressure.

### Prognostic impact of J-MACS score

Patients were followed for a median of 514 days (IQR: 257-730 days) after LVAD implantation. During the observation period, 19 patients died, meeting the primary endpoint. The J-MACS score, analyzed as a continuous variable, was independently associated with the primary outcome, with a hazard ratio of 1.17 (95% CI: 1.02-1.36, *P* = 0.037) after adjustment for other potential confounders (Table 2). The proportional hazards assumption was not violated for the variables included in the models (all *P* > 0.05). Calibration analysis demonstrated good agreement between predicted and observed mortality across deciles, with a nonsignificant Hosmer-Lemeshow statistic (chi-square = 6.21, df = 8, *P* = 0.62). The observed-to-expected ratio was 1.16 (95% CI: 0.73-1.81), the calibration slope was 1.00 (95% CI: 0.09-1.91), and the calibration plot demonstrated overall acceptable agreement between predicted and observed 2-year mortality (Supplemental Figure 1). There was no significant interaction between age >65 years and the prognostic effect of the J-MACS score (*P*_interaction_ = 0.45).

**Table 2:** Potential Baseline Predictors for the Primary Outcome

	Univariable Analysis		Multivariable Analysis	
	HR (95% CI)	*P* Value	HR (95% CI)	*P* Value
Male	1.22 (0.45-3.31)	0.60	—	—
INTERMACS profile 1 or 2	1.06 (0.41-2.45)	0.91	—	—
Hemoglobin	0.88 (0.64-1.12)	0.25	—	—
Serum albumin	0.71 (0.35-1.45)	0.41	—	—
Plasma BNP	1.04 (0.31-3.42)	0.94	—	—
Left ventricular end-diastolic diameter	0.71 (0.49-1.09)	0.085	0.71 (0.45-1.11)	0.14
Moderate or greater mitral regurgitation	0.95 (0.40-2.31)	0.76	—	—
Moderate or greater tricuspid regurgitation	2.54 (1.07-6.23)	0.032^a^	1.23 (0.45-3.40)	0.69
Intravenous inotrope use	2.73 (0.64-11.5)	0.19	—	—
J-MACS score	1.18 (1.09-1.28)	<0.001^a^	1.17 (1.02-1.36)	0.037^a^

The prognostic impact of the potential predictors, including J-MACS score as a continuous variable, was evaluated by Cox proportional hazard ratio regression analysis. All variables were preselected among baseline characteristics. Variables with *P* < 0.10 in the univariable analyses were included in the multivariable analysis with a forced method.

BNP = B-type natriuretic peptide.

a *P* < 0.05.

In Fine-Gray subdistribution hazard analysis treating device explantation (n = 6) as a competing event, the J-MACS score was significantly associated with mortality (subdistribution HR: 1.18 per point increase, 95% CI: 1.02-1.38, *P* = 0.029).

Using Rubin's rules to combine estimates across the 5 multiply imputed datasets, the J-MACS score was significantly associated with mortality, with a hazard ratio of 1.15 (95% CI: 1.02-1.41, *P* = 0.024).

The Harrell's C-statistic for the J-MACS score was 0.66 (95% CI: 0.49-0.70) (Figure 3). A cutoff value of 10.2 yielded a sensitivity of 79% (95% CI: 57%-92%) and a specificity of 53% (95% CI 46%-60%), while a cutoff of 13.7 yielded a sensitivity of 37% (95% CI 19%-59%) and a specificity of 90% (95% CI 84%-93%). Based on these cutoffs, the entire cohort was categorized into low-, intermediate-, and high-risk groups. The apparent C-statistic was 0.66, and the estimated optimism was 0.03, yielding an optimism-corrected C-statistic of 0.63.

**Figure 3:**
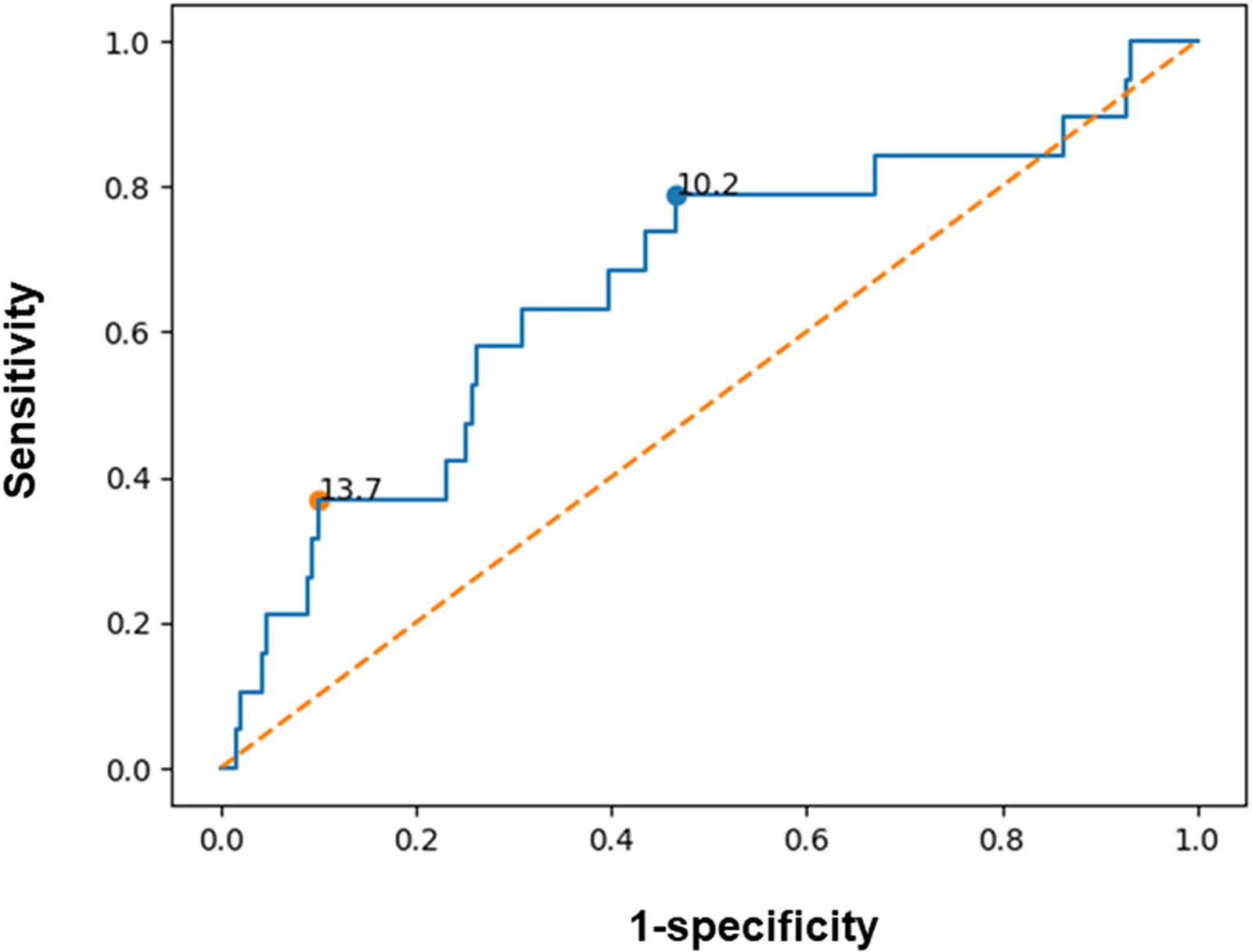
Receiver Operating Characteristic Curve of the J-MACS Score The area under the curve was 0.66 (95% CI: 0.53-0.79). Sensitivity and specificity for the predefined cutoffs of 10.2 and 13.7 are displayed as red circles.

### Prognostic impact of risk-group stratification

The hazard ratio for the intermediate-risk group compared with the low-risk group was 2.75 (95% CI: 0.83-9.14, *P* = 0.099), and for the high-risk group compared with the low-risk group, it was 6.81 (95% CI: 1.99-23.3, *P* = 0.002). The 2-year cumulative mortality rates were 5.8% (95% CI: 2.0%-16.1%) in the low-risk group, 12.7% (95% CI: 6.3%-24.6%) in the intermediate-risk group, and 28.6% (95% CI: 14.6%-51.2%) in the high-risk group (*P* = 0.003 for comparison) (Figure 4). The RMST at 2 years was 709.4 days in the low-risk group, 669.6 days in the intermediate-risk group, and 592.7 days in the high-risk group (Figure 5). The RMST difference analysis further demonstrated a clinically meaningful loss of approximately 4 months in the high-risk group compared with the low-risk group (RMST difference +117 days; 95% CI: 24-217 days, *P* = 0.007), whereas the difference between the low- and intermediate-risk groups showed only a borderline trend (40 days; 95% CI: −6 to 90 days, *P* = 0.10).

**Figure 4:**
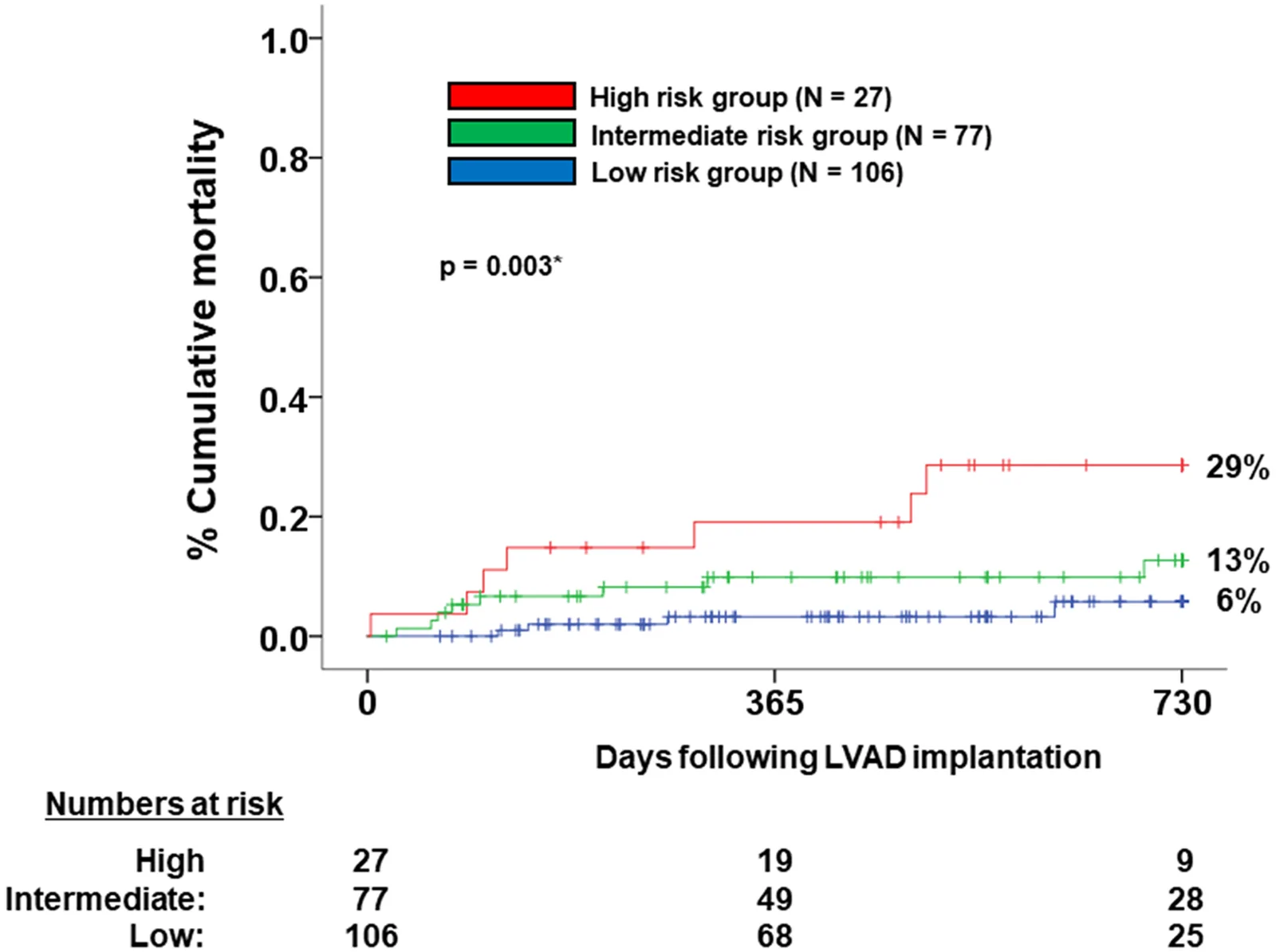
Kaplan-Meier Curves for 2-Year Mortality Stratified by J-MACS Risk Categories Mortality progressively increased from low- to high-risk groups (6%, 13%, and 29%, respectively). Numbers at risk are shown below. LVAD = left ventricular assist device. ∗*P* < 0.05 by log-rank test.

**Figure 5:**
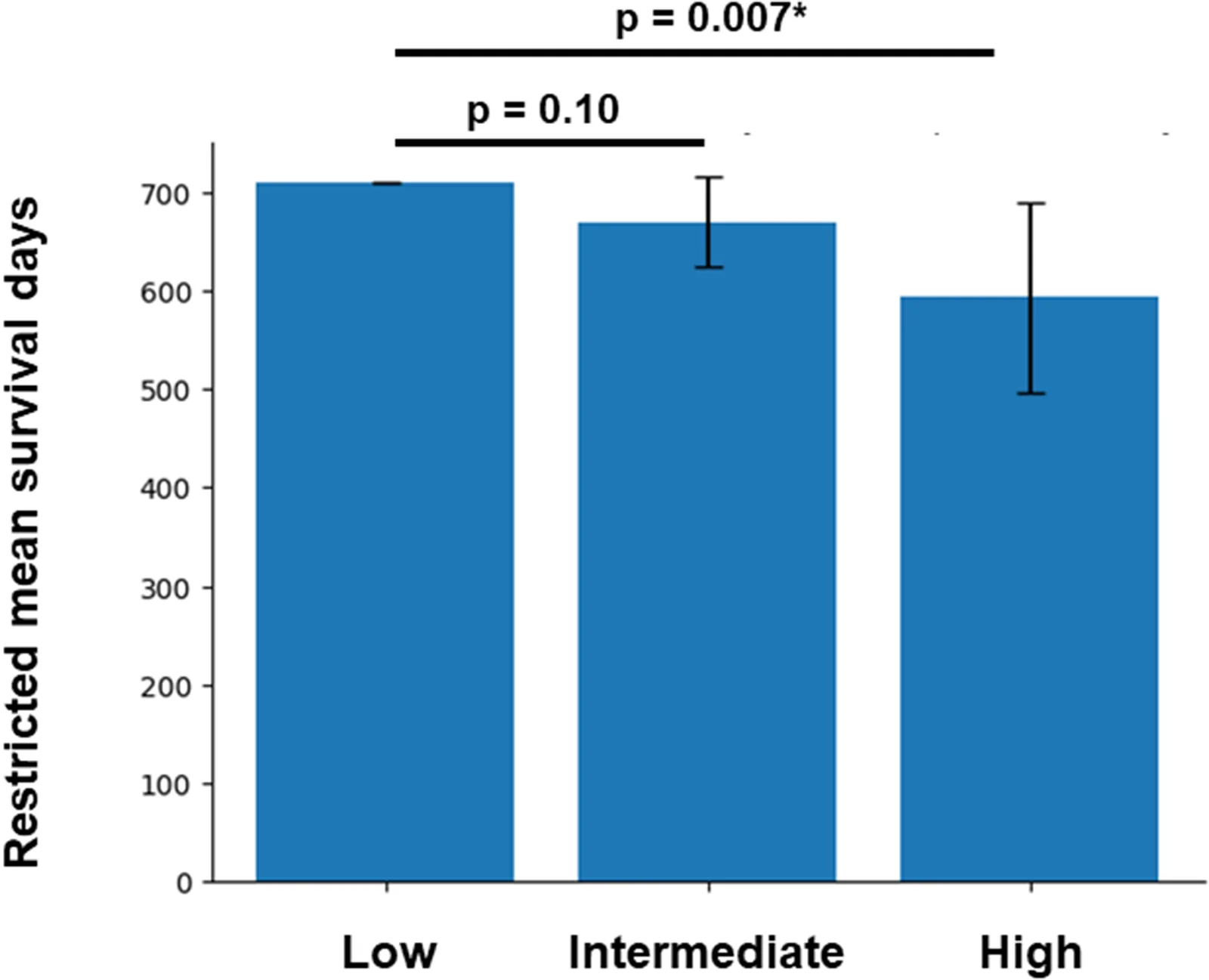
Restricted Mean Survival Time Over 2 Years According to J-MACS Risk Group Restricted mean survival day was significantly shorter in high-risk patients compared with low-risk patients (*P* = 0.007), whereas the difference between the intermediate-risk and low-risk groups was borderline (*P* = 0.10). ∗*P* < 0.05 by nonparametric bootstrap.

### Comparison with other risk scores

The median HMII-RS was 1.32 (IQR: 0.67-1.87). When analyzed as a continuous variable, the HMII-RS was not significantly associated with the primary outcome (unadjusted HR: 1.31, 95% CI: 0.85-2.04, *P* = 0.23). Using predefined cutoffs of 1.58 and 2.48, 128 patients were classified into the low-risk group, 57 into the intermediate-risk group, and 25 into the high-risk group. These groups did not significantly differ in terms of the primary outcome (*P* = 0.87).

The median HM3-RS was 2.55 (IQR: 2.14-2.88). When treated as a continuous variable, the HM3-RS was significantly associated with the primary outcome (unadjusted HR: 2.30, 95% CI: 1.07-4.96, *P* = 0.033). Using cutoffs of 2.41 and 2.97, 88 patients were categorized as low risk, 80 as intermediate risk, and 42 as high risk. However, 2-year mortality was not significantly stratified among these groups (*P* = 0.38).

The Harrell's C-statistic of J-MACS was significantly higher than that of HMII-RS (0.58, 95% CI: 0.45-0.70, *P* = 0.025) and trended to be higher than that of HM3-RS (0.62, 95% CI: 0.48-0.76, *P* = 0.086). Decision curve analysis demonstrated that the J-MACS score provided comparable net benefit to other scores across lower threshold probabilities and a tendency toward greater net benefit at higher threshold probabilities (Supplemental Figure 2). In continuous reclassification analysis, the J-MACS score demonstrated modest reclassification improvement over the HMII-RS (NRI: +0.45, IDI: +0.0023) and the HM3-RS (NRI: +0.36, IDI: +0.0015).

## Discussion

### Principal findings

In this nationwide registry–based external validation study including all Japanese destination therapy patients with HeartMate 3 LVAD, we demonstrated that the J-MACS score, which is mandatory to be calculated in Japan before destination therapy, was independently associated with 2-year mortality and successfully stratified patients into distinct prognostic categories (Central Illustration). RMST analysis showed approximately 4 months shorter survival in the high-risk group compared with the low-risk group. Calibration was acceptable, its risk stratification ability was superior to other scorings, and no significant interaction with age >65 years was observed, suggesting consistent performance across age categories.

**Central Illustration:**
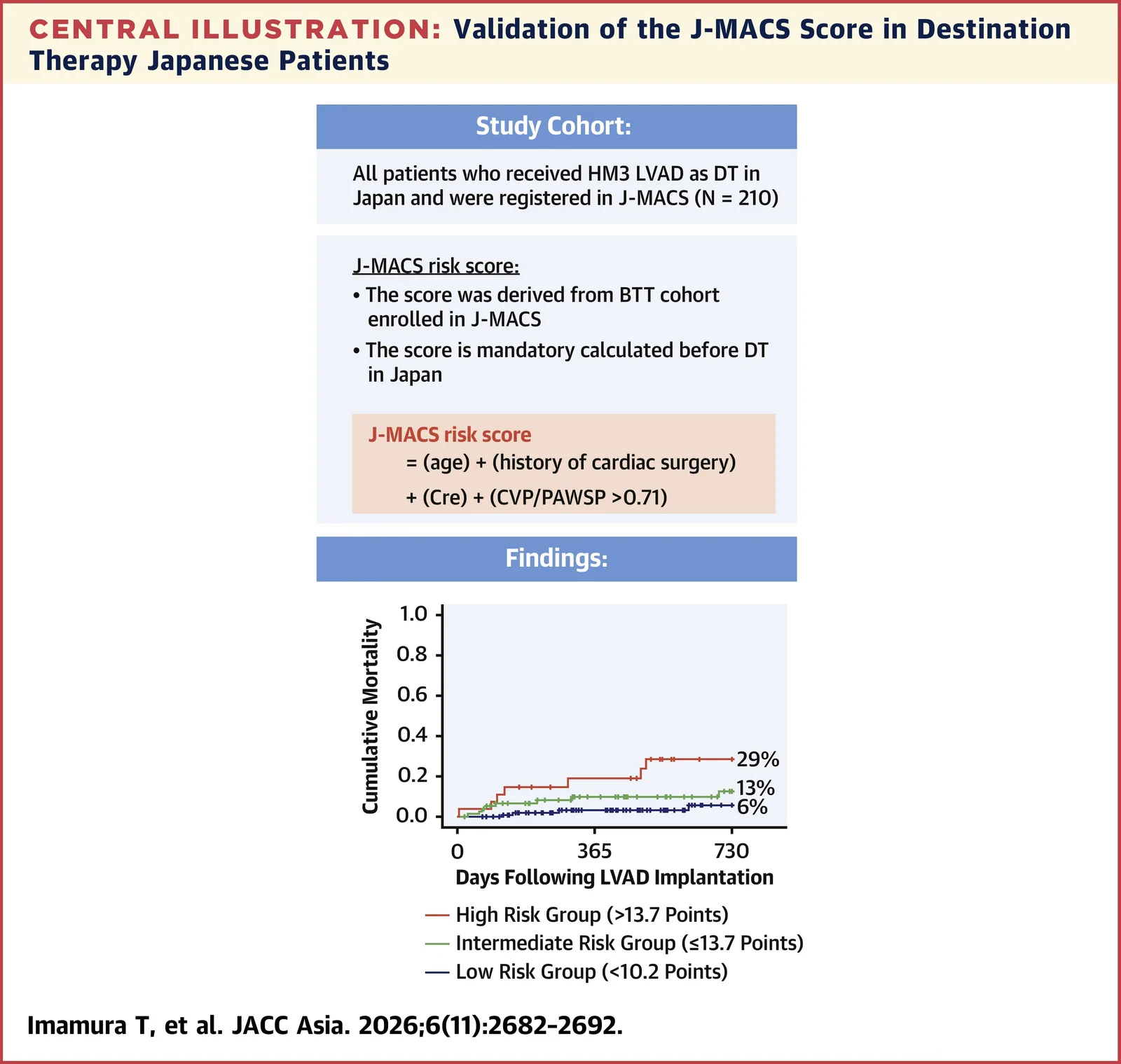
Validation of the J-MACS Score in Destination Therapy Japanese Patients BTT = bridge to transplantation; Cre = creatinine; CVP = central venous pressure; DT = destination therapy; HM3 = HeartMate 3; LVAD = left ventricular assist device; PAWP = pulmonary artery wedge pressure.

### Clinical and policy implications

Unlike conventional validation studies, this analysis evaluates a risk model already embedded within a nationally mandated clinical decision framework. Because J-MACS scoring is required before destination therapy implantation in Japan, our findings extend beyond statistical validation to real-world assessment of a currently existing treatment algorithm. Despite moderate discrimination metrics, predefined risk categories showed meaningful survival separation, including significant differences in restricted mean survival time, supporting the role of the J-MACS score as a structured tool for multidisciplinary decision-making and patient counseling.

### Comparison with other previous scores

Several risk prediction models, including the HMII-RS and HM3-RS, have been developed in Western cohorts.^8^^,^^9^ However, in the present study, neither score showed sufficient discriminatory performance to stratify 2-year mortality among the Japanese destination therapy cohort. The J-MACS score, which was originally developed from Japanese LVAD recipients listed for transplant candidacy,^12^ contains only 4 simple baseline variables, yet demonstrated stronger discrimination in this Japanese destination therapy cohort compared with the other risk scores.

Center volume is one of the main contributors to the HMII-RS.^8^ However, destination therapy centers are nationally certified, and no institute satisfied the item of “smaller center volume” in the present study. In the contemporary era, we can treat/correct hyponatremia, which was one of the important items for calculating the HM3-RS,^9^ by adjusting the dose of vasopressin type-2 antagonist.^15^ These inappropriate items might have reduced the predictability of these conventional scores.

### Validity of the J-MACS score in the destination therapy cohort

Age is included in the J-MACS score because it is a key determinant of destination therapy candidacy in Japan, where age over 65 years remains a contraindication for heart transplantation listing.^6^ The J-MACS score was applicable irrespective of age in the present study, with a statistically nonsignificant interaction.

Although many forms of end-organ dysfunction can be ameliorated after HeartMate 3 implantation when present at only a moderate degree, right ventricular failure and renal impairment still represent the major unresolved issues.^16^^,^^17^ These complications are particularly critical among destination therapy recipients, who require long-term durable LVAD support and consequently experience morbidity and mortality.^18^ Therefore, both the CVP/PAWP ratio and serum creatinine—parameters that directly reflect right-sided congestion and renal reserve—serve as clinically relevant contributors to risk stratification within the J-MACS score.

Another important item in the J-MACS score is a history of cardiac surgery. A prior sternotomy is associated with more complex intraoperative management, including prolonged cardiopulmonary bypass and cross-clamp times, greater operative bleeding, and a higher likelihood of postoperative right ventricular failure, all of which adversely affect long-term outcomes.^19^

### Practical implications of J-MACS score

Because J-MACS scoring is mandatory in Japan, our findings provide direct validation of a nationally implemented destination therapy eligibility framework. Given that the contemporary 3-year mortality of bridge-to-transplant LVAD candidates in Japan is approximately 12%, destination therapy may be reasonably considered in patients classified as low or intermediate risk, whose 2-year mortality was 6% and 13%, respectively. Risk-based stratification using the J-MACS score may support more personalized management strategies, including optimization of right heart function, renal management, and postoperative monitoring.^20^

### Future perspectives

Future studies should focus on refining and externally validating the J-MACS score in larger and more diverse destination therapy populations. Integration with biomarkers, echocardiographic parameters, and frailty assessments may improve predictive performance. Furthermore, incorporation into dynamic, data-driven risk platforms using longitudinal registry data could enable adaptive risk assessment and support real-time clinical decision-making. Conversely, complex risk scorings may lose the simplicity of their calculation.

### Study Limitations

First, the retrospective design and relatively small sample size may limit generalizability, although this was a nationwide Japanese cohort. Second, residual confounding cannot be completely excluded despite adjustment for potential covariates, given small event numbers and restricted numbers of included variables in the multivariable model. Third, the number of adverse events was modest, and the median follow-up period was shorter than the intended 2 years for some patients. Fourth, device management protocols were not standardized and may have varied across centers. Fifth, patients classified as high risk according to the J-MACS score were generally not considered candidates for destination therapy under current clinical practice in Japan. This likely resulted in an under-representation of the highest risk patients in the present cohort, which may have attenuated the observed discriminatory performance. Nonetheless, the J-MACS score demonstrated clear separation in Kaplan-Meier and RMST analyses, supporting its clinical utility for risk stratification.

## Conclusions

The J-MACS score provides meaningful risk stratification among Japanese destination therapy recipients undergoing HeartMate 3 implantation. As a nationally mandated decision tool, its real-world validation supports current risk-based patient selection and reinforces its role in guiding clinical decision-making and health care resource allocation in Japan.

### Ethics Statement

The present study was approved by the J-MACS Committee (reference number: 2203).

## Funding Support and Author Disclosures

Dr Sakata has received honoraria from Abbott. All other authors have reported that they have no relationships relevant to the contents of this paper to disclose.
