# Supplementary material for: Real-World Evaluation of a Nationally Mandated Risk Stratification Tool for Destination Therapy LVAD in Japan
Source: JACC Asia. 2026 Jul 24;6(11):2682–92. doi: 10.1016/j.jacasi.2026.06.017 (PMC13750888; doi:10.1016/j.jacasi.2026.06.017)
Supplement: Supplementary file 1 [file mmc1.docx]

Supplementary Figure 1. Calibration plot for 2-year mortality

Calibration was assessed by comparing predicted and observed 2-year mortality across deciles of predicted risk. Observed event rates were estimated using Kaplan–Meier methods. The solid line represents the ideal line of perfect calibration (45°), and the dashed line represents the observed relationship between predicted and observed risks. Overall, the model demonstrated acceptable agreement, although some variability was observed in higher-risk strata due to the limited number of events.

Supplementary Figure 2. Decision curve analysis comparing the clinical utility of the J-MACS score with other scores

Net benefit is plotted against threshold probability. The J-MACS score demonstrated comparable net benefit to alternative models across lower threshold probabilities, with a tendency toward greater net benefit at higher threshold ranges.
